# Comparative study of serum proteomes in Legg-Calve-Perthes disease

**DOI:** 10.1186/s12891-015-0730-z

**Published:** 2015-10-05

**Authors:** Ruiyu Liu, Lihong Fan, Longbin Yin, Kunzheng Wang, Wusheng Miao, Qichun Song, Xiaoqian Dang, Hang Gao, Chuanyi Bai

**Affiliations:** Department of Orthopaedic, the Second Hospital Affilicated to Medical College, Xi’an Jiaotong University, No.157, Xiwu Road, Xi’an, Shaanxi 710004 P. R China; Department of Pediatric Orthopaedic, Hong-Hui Hospital, Medical College of Xi’an Jiaotong University, No. 76 Nanguo Road, Nanshao Men, Beilin District, Xi’an, 710054 P. R China; Research Center for Proteome Analysis, Shanghai Institutes for Biological5 Sciences, Chinese Academy of Sciences, Shanghai, P. R China

**Keywords:** Legg-Calve-Perthes disease, Serum proteome, Isobaric tags for relative and absolute quantification, Lipid metabolism

## Abstract

**Background:**

Legg-Calve-Perthes Disease (LCPD) is an idiopathic osteonecrosis of the developing femoral head complicated by pain and disability of the hip joint. To date, the pathological mechanisms of LCPD are not well-known. This study screened the changes in serum protein expression in patients with LCPD.

**Methods:**

Age- and sex-matched serum samples from 10 control subjects and 10 patients with LCPD were compared using the isobaric tags for relative and absolute quantification (iTRAQ) technique. Gene ontology analyses, KEGG pathway and functional network analyses were performed. Proteins of interest with large differences in expression, S100-A8, alpha-1-acid glycoprotein 1, haptoglobin and apolipoprotein E, were compared by western blotting.

**Results:**

The disease/control ratios showed 26 proteins were significantly differentially expressed (all *p* < 0.05). Including higher abundances of complement factor H (1.44), complement C4-B (1.45), isocitrate dehydrogenase [NAD] subunit alpha (2.7) alpha-1-acid glycoprotein 1 (1.87), heptoglobin (1.53) and Ig lambda-2 chain C regions (1.46), and lower levels of apolipoprotein E (0.50), apolipoprotein F (0.60), apolipoprotein C-III (0.69), S100-A8 (0.73), S100-A9 (0.75) and prothrombin (0.77) in LCPD than in controls. The alpha-1-acid glycoprotein 1 and haptoglobin increases, and apolipoprotein E and S100-A8 decreases were confirmed by western blot. KEGG pathway analysis revealed these proteins were related to the complement and coagulation cascades, *Staphylococcus aureus* infection, PPAR signaling, fat digestion and absorption, and vitamin digestion and absorption. Functional network analysis suggested that the proteins were involved in lipid regulation.

**Conclusions:**

The complement and coagulation cascades, and abnormal lipid metabolism may be involved in the pathogenesis of LCPD.

## Background

Legg-Calve-Perthes disease (LCPD) is an idiopathic osteonecrosis of the developing femoral head complicated by pain and disability of the hip joint. The disease affects childhood between 6 and 9 years of age. The estimated annual incidence ranges from 0.2 to 19.1 per 100,000 among children under 15 years [1]. It occurs 5 times more often in boys than in girls [2]. The main long-term problem with this disease is a permanent deformity of the femoral head, which increases the risk of developing osteoarthritis in adults [3]. However, the underlying mechanisms and determinants of LCPD are still unclear including the proteins involved. This situation hampers effective treatment and etiological investigation.

Coagulation abnormalities [4–6], vascular interruptions of the blood supply to the proximal femur [7] and abnormal immunological reactivity [8] are all linked to the pathogenesis of LCPD. In addition, it has been suggested that leptin, a lipid regulator, might play an important role [9]. Another study showed abnormalities in the vascular structure and function in children with LCPD disease [10]. In experimental animal models, acute bone morphogenic protein 2 (BMP2) upregulation [11], increased vascular endothelial growth factor (VEGF) expression in the epiphyseal cartilage [12], and increased matrix mineralization [13] in the immature femoral head follow induction of ischemic osteonecrosis. These findings imply that multiple pathological reactions may be involved in the development of LCPD.

Comparative serum proteomic analysis has been used for years as a potent tool for understanding the pathology of many diseases, especially for those diseases, like LCPD, about which we have very limited knowledge. A similar strategy has been applied to osteonecrosis of the femoral head in adults [14, 15]. Given that these two diseases occur in the same location and have similar symptoms the results of that study should also help us understand the pathology of LCPD. Isobaric tags for relative and absolute quantitation (iTRAQ), is a quantitative proteomic approach with relatively high throughput that allows for simultaneous identification and peptide quantification by measuring the peak intensities of reporter ions with tandem mass spectroscopy (MS/MS) that has been developed and utilized to identify biomarkers for various disease conditions [16, 17]. This chemical labeling method involves the stable incorporation of isotopes into an amine tagging reagent, which can then be reliably detected by mass spectrometry, thereby permitting comparative quantitation of various proteins. This method has been suggested to be suitable for the discovery of differentially expressed proteins in a wide range of body fluids and tissues, including serum and plasma [17, 18].

In this study, we performed a comparison of serum proteomes in child patients with LCPD using the bioinformation match iTRAQ method, to assess the simultaneous expression of several serum proteins. This allowed us to characterize the associations between differentially expressed proteins and the incidence of LCPD.

## Methods

### Patients

The study included a total of 20 healthy children and 20 patients with LCPD. All the patients and the gender/age matched healthy children were recruited between November 2011 and August 2012 from the second Hospital affiliated to Xian Jiaotong University (Xi’an, China). The diagnosis of LCPD was verified based upon the radiographic appearance and clinical features [19]. The recruited patients were diagnosed with the disease for the first time and they had accepted no treatment including agent, surgery or the other treatments (non-weight bearing, bed rest, cast, etc.) before their blood was sampled. Cases of multiple epiphyseal dysplasia, cerebral palsy, and developmental hip dysplasia were excluded owing to their known independent association with avascular necrosis of the hip. The severity of the disease was evaluated according to the Catterall staging system [19], there were four stage I patients, five stage II patients and one stage III patients. The healthy children, who had a physical examination before admission to our hospital, were randomly enrolled to serve as the control group in the same period by being age- and gender-matched. The recruitment criteria was as follows: the healthy children had no disease and bone disease history, with no previous or current symptoms related to the hip and a normal hip radiograph (if available) or no limitation of hip abduction and internal rotation (in children without hip radiographs). There were no significant differences in body weight and height between the two groups. ITRAQ analysis was performed for the first ten patients and ten healthy children (clinical characteristics are showed in Table 1). The blood sample of another 20 patients and the age and sex matched healthy children were used to performed west-blotting analysis (clinical characteristics are shown in Table 2).

**Table 1:** Clinical characteristics in two groups for iTRAQ analysis

Group	Patient group	Healthy group
Sex (female /male)	1/9	1/9
Age (Years)	5.4 ± 1.9	5.5 ± 2.0
Height (cm)	111.5 ± 14.9	109.6 ± 15.1
Weight (kg)	20.5 ± 5.1	20.9 ± 4.8

**Table 2:** Clinical characteristics in two groups for West-blotting verification

Group	Patient group	Healthy group
Sex (female /male)	3/17	3/17
Age (Years)	5.6 ± 2.1	5.8 ± 2.3
Height (cm)	113.5 ± 12.3	108.6 ± 16.3
Weight (kg)	22.5 ± 5.4	21.9 ± 5.0

This study was approved by the Ethical Committee of the Second Affiliated Hospital of Xi’an Jiaotong University, Xi’an, China. Signed informed-consent documents were obtained from all the study participants and their guardians.

### Protein Digestion and isobaric tags for relative and absolute quantification (iTRAQ) labeling

Two milliliters of peripheral venous blood was obtained from each patient in the outpatient department, and processed to collect serum and then stored at −80 °C for further analysis. Fourteen high-abundance proteins in the serum pools were depleted using a multiple affinity removal system (Agilent Human 14, Agilent Technologies, USA) following the manufacturer’s protocol. A 5 KDa ultrafiltration tube (Sartorius) was used for desalination and concentration. The protein content of the depleted serum was determined by the Bradford assay (Bio-Rad protein assay) according to the manufacturer’s instructions.

Protein digestion was performed according to the Filter aided sample preparation (FASP) procedure described by Wisniewski et al. [16]. The resulting peptide mixture was labeled using the 4-plex iTRAQ reagent (Applied Biosystems). Briefly, 200 μg of peptides from each sample were added to 30 μl of STD buffer (4 % sodium dodecyl sulfate (SDS), 100 mM dithiothreitol (DTT), 150 mM Tris–HCl pH 8.0). The detergent, DTT and other low-molecular-weight components were removed using UA buffer (8 M Urea, 150 mM Tris–HCl pH 8.0) by repeated ultrafiltration (Microcon units, 30 kD). Then 100 μl 0.05 M iodoacetamide in UA buffer was added as a blocking agent and the samples were incubated for 20 min in darkness. The filters were washed with 100 μl of UA buffer three times and then 100 μl DS buffer (50 mM triethylammoniumbicarbonate at pH 8.5) twice. Finally, the protein suspensions were digested with 2 μg of trypsin (Promega, Madison, USA) in 40 μl of DS buffer overnight at 37 °C, and the resulting peptides were collected as a filtrate. The peptide content was estimated by UV light spectral density at 280 nm using an extinction coefficient of 1.1 of 0.1 % (g/l) solution that was calculated on the basis of the frequency of tryptophan and tyrosine in vertebrate proteins [20, 21].

For labeling, each iTRAQ reagent was dissolved in 70 μl of ethanol and added to the respective peptide mixture. The samples were labeled as Serum1-114, Serum2-115, Serum3-116, and Serum4-117, and were multiplexed and vacuum dried.

### Mass Spectrum (MS) analyses

The peptide mixture was desalted on C18 Cartridges (Empore™ SPE Cartridges C18, standard density, bed I.D. 7 mm, volume 3 ml, Sigma, St. Louis, USA), then concentrated by vacuum centrifugation and reconstituted in 40 μl of 0.1 % (v/v) trifluoroacetic acid. MS experiments were performed on a Q Exactive mass spectrometer that was coupled to Easy nLC (Proxeon Biosystems, now Thermo Fisher Scientific). 10 μl of the sample was injected for nanoLC-MS/MS analysis. The peptide mixture (5 μg) was loaded onto a C18-reversed phase column (15 cm long, 75 μm inner diameter) packed in-house with RP-C18 5 μm resin in buffer A (0.1 % Formic acid) and separated with a linear gradient of buffer B (80 % acetonitrile and 0.1 % Formic acid) at a flow rate of 250 nl/min controlled by IntelliFlow technology over 240 mins. MS data was acquired using a data-dependent top10 method dynamically choosing the most abundant precursor ions from the survey scan (300–1800 m/z) for HCD (higher energy collisional dissociation) fragmentation. Determination of the target value was based on predictive automatic gain control (pAGC). Dynamic exclusion duration was 60 s. Survey scans were acquired at a resolution of 70,000 at m/z 200 and resolution for HCD spectra was set to 17,500 at m/z 200. Normalized collision energy was 30 eV and the underfill ratio, which specifies the minimum percentage of the target value likely to be reached at maximum fill time, was defined as 0.1 %. The instrument was run with peptide recognition mode enabled.

### Protein identification

MS/MS spectra were searched using MASCOT engine (Matrix Science, London, UK; version 2.2) embedded into Proteome Discoverer 1.3 (Thermo Electron, San Jose, CA.) against UniProt Human database (133549 sequences, downloaded on March 3rd, 2013) and the decoy database. For protein identification, the following options were used. Peptide mass tolerance = 20 ppm, MS/MS tolerance = 0.1 Da, Enzyme = Trypsin, Missed cleavage = 2, Fixed modification: Carbamidomethyl (C), iTRAQ4plex (K), iTRAQ4plex (N-term).

## Gene ontology analyses

The gene ontology (GO) method is a major bioinformatics initiative with the aim of standardizing the representation of protein attributes across species and databases. The online system provides a controlled vocabulary of terms for describing gene product characteristics and gene product annotation data from GO consortium members, as well as tools to access and process this data. Each protein is characterized in terms of three ontologies: molecular function, cellular component and the involved biological process. Using the GO database (http://www.geneontology.org/) and Onto-Express analysis [18], the involved genes were classified in order to gain an overall picture of potential functions of the differentially expressed genes in this study.

### KEGG pathway analyses

The orthology of the proteins was investigated using the Kyoto encyclopedia of genes and genomes (KEGG) database, by adopting a web-based server called KAAS (KEGG Automatic Annotation Server: http://www.genome.jp/kegg/kaas/). The proteins were annotated with the KEGG orthology (KO) identifiers, or the K numbers, based on the best hit information using Smith–Waterman scores as well as by the manual curation. Each K number represents an ortholog group of genes, and it is directly linked to an object in the KEGG pathway map or the BRITE functional hierarchy. The method is based on sequence similarities, bi-directional best hit information and some heuristics, and has achieved a high degree of accuracy when compared with the manually curated KEGG GENES database.

### Functional network analyses

To investigate the direct (physical) and/or indirect (functional) interactions among the identified genes, we further utilized the search tool for the retrieval of interacting genes (STRING) database (http://string.embl.de/) to analyze the functional network. The STRING database provided a score for each gene-gene interaction, which is computed as the joint probability of the probabilities from the different evidence channels (protein interaction, fusion, co-expression, text mining, etc.), correcting for the probability of randomly observing an interaction [22]. A high database score meant that there were high experimental or predicted evidence for gene-gene functional interaction. Therefore a functional associated network was constructed based on the differential expression profile in the present study.

### Western blotting analysis

Commercial antibodies used for western blotting validation experiments were as follows: haptoglobin (HP) (31083) (Signalway Antibody LLC, Baltimore, USA), S100-A8 (ab92331), and orosomucoid 1 (ORM1) (ab133642), apolipoprotein E (APOE) (ab52607) and actin monoclonal antibodies (all from Abcam, Cambridge, MA, USA). Processed serum from another 30 adolescents (15 LCPD patients and 15 age and sex matched healthy adolescents) that had undergone depletion of high-abundance proteins was used for western blotting analysis. SDS polyacrylamide gel electrophoresis (SDS-PAGE) was performed on 12-15 % acrylamide gels with 60 μg protein per lane, and proteins were electrophoretically transferred onto nitrocellulose membranes (Millipore, Billerica, MA, USA). Membranes were blocked with blocking buffer purchased from LICOR (Lincoln, NE, USA) and then incubated overnight at 4 °C with primary antibody. The dilutions were performed as suggested by the manufacturers. The membranes were washed with phosphate buffered saline (PBS) followed by incubation with a horseradish peroxidase-conjugated secondary antibody (Santa Cruz Biotechnology, Inc., Santa Cruz, CA, USA) at 37 °C for 2 hours, and then chemiluminescence detection by ECL plus. Captured images were analyzed using a LAS4000 (Fuji Film) and Multi Gaugev2.0 (Fuji Film). For quantification of western blotting data, each protein signal was quantitated and normalized by the internal protein actin intensity.

### Statistical analysis

The statistical significance of the differential expression levels on proteins was tested by *t*-test. A protein with a disease/healthy control ratio > 1.2 and *p* <0.05 was considered as significant. The method of false discovery rate (FDR) was used for addressing the multiple-testing adjustment, and adjusted *p* < 0.01 was used as the significant criterion.

## Results

### Protein identification

**Fig. 1:**
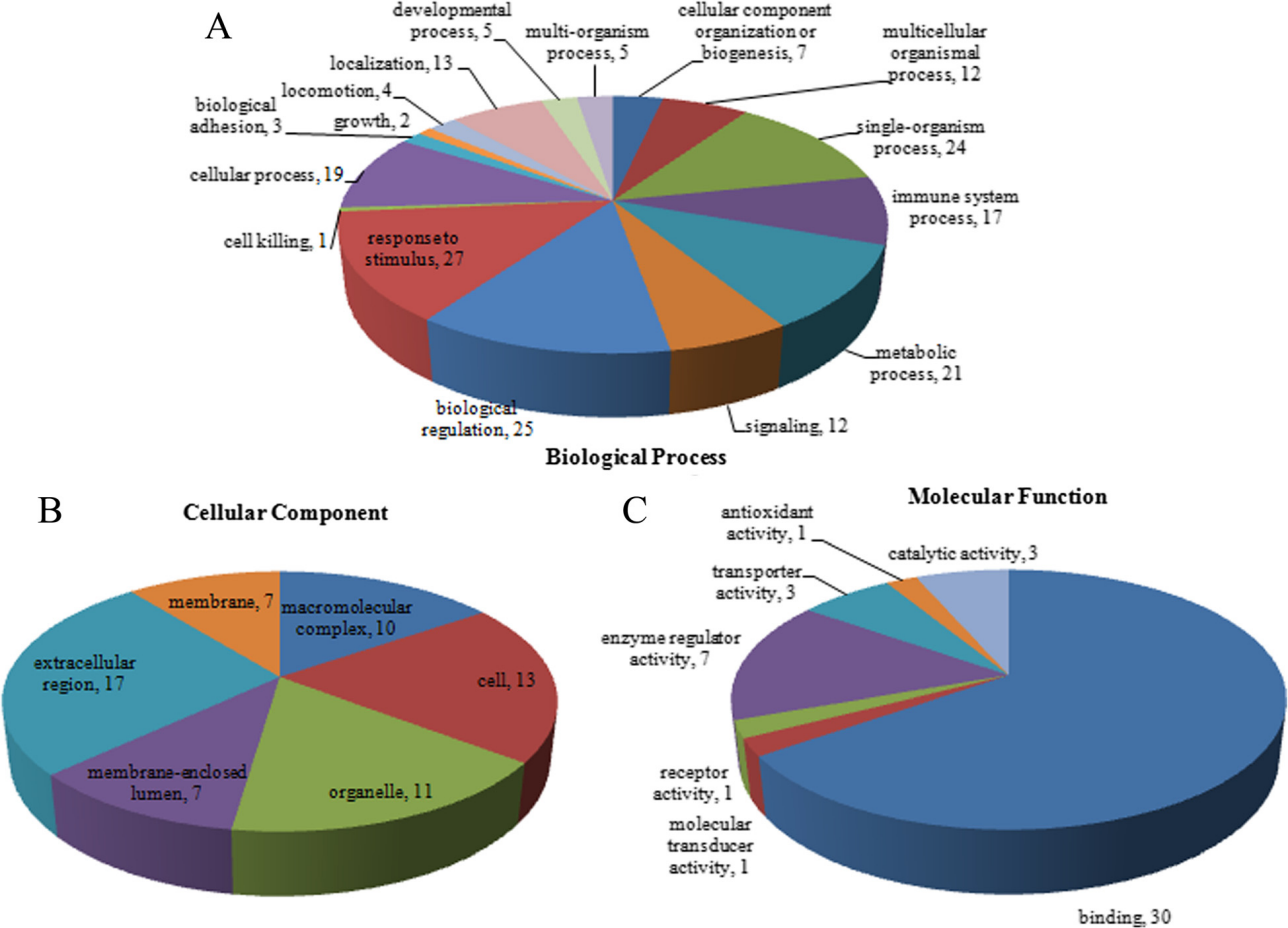
Protein classification. **a** based on the ontology of biological processes; **b** based on the ontology of cellular components; **c** based on the ontology of molecular functions

We identified 223 protein groups from a total of 2803 unique peptides. All the identified proteins were classified using the GO annotation (http://david.abcc.ncifcrf.gov/home.jsp) and further categorized into three functional groups: biological processes (Fig. 1a), cellular components (Fig. 1b) and molecular functions (Fig. 1c).

The GO analyses showed that the most common molecular function was binding activity, which included ion binding, protein binding, carbohydrate binding, pattern binding, cell surface binding, and lipid binding. Another major functional category was enzyme regulation. The most frequently encountered biological processes were biological regulation, response to stress, localization, and establishment of localization.

### Differentially expressed proteins

There was a significant difference of expression of 26 proteins that had specific functions between the disease and control groups (shown in Table 3). Complement activation related proteins, complement C4-B and complement factor H, were more highly expressed in the disease group. However, the cartilage metabolism related protein S100-A8/S100-A9 was reduced in the disease group. Furthermore, we found that the immune response related protein, Ig kappa chain C region and Ig lambda-2 chain C region, had significant increased expression in LCPD. And prothrombin, which has a close relationship with coagulation, was lower in the disease group.

**Table 3:** Differentially Expressed Proteins in LCPD and Control Groups

Accession no.	Protein description	Gene name	Disease/control Ratio	*p*-value
Q8TCZ8	Apolipoprotein E	APOE	0.50	<0.001
F5GXS5	Apolipoprotein F	APOF	0.60	<0.001
B7Z539	Inter-alpha-trypsin inhibitor heavy chain H1	ITIH1	0.67	<0.001
P02656	Apolipoprotein C-III	APOC3	0.69	<0.001
P05109	S100-A8	S100A8	0.73	0.004
P01860	Ig gamma-3 chain C region	IGHG3	0.74	0.006
Q59HB3	Apolipoprotein B	APOB	0.74	0.007
B2R4M6	S100-A9	S100A9	0.75	0.008
P02655	Apolipoprotein C-II	APOC2	0.76	0.010
P00734	Prothrombin	F2	0.77	0.017
B2R701	Peptidase inhibitor 16	PI16	0.79	0.027
P11226	Mannose-binding protein C	MBL2	0.79	0.030
B7Z544	Inter-alpha-trypsin inhibitor heavy chain H4	ITIH4	0.80	0.040
P36980	Complement factor H-related protein 2	CFHR2	0.80	0.470
B3KWB5	Alpha-1B-glycoprotein	A1BG	1.288	0.033
P01859	Ig gamma-2 chain C region	IGHG2	1.315	0.020
A8K5T0	Complement factor H	CFH	1.44	0.002
B7Z1F8	Complement C4-B	C4B	1.45	0.001
P0CG05	Ig lambda-2 chain C regions	IGLC2	1.46	0.001
A2J1M4	Rheumatoid factor RF-ET7 (Fragment)		1.46	0.001
P00738	Haptoglobin	HP	1.53	<0.001
P01834	Ig kappa chain C region	IGKC	1.60	<0.001
P02763	Alpha-1-acid glycoprotein 1	ORM1	1.87	<0.001
P01009	Alpha-1-antitrypsin	SERPINA1	1.93	<0.001
P02647	Apolipoprotein A-I	APOA1	2.0	<0.001
H0YLI6	Isocitrate dehydrogenase [NAD] subunit alpha, mitochondrial	IDH3A	2.7	<0.001

**Fig. 2:**
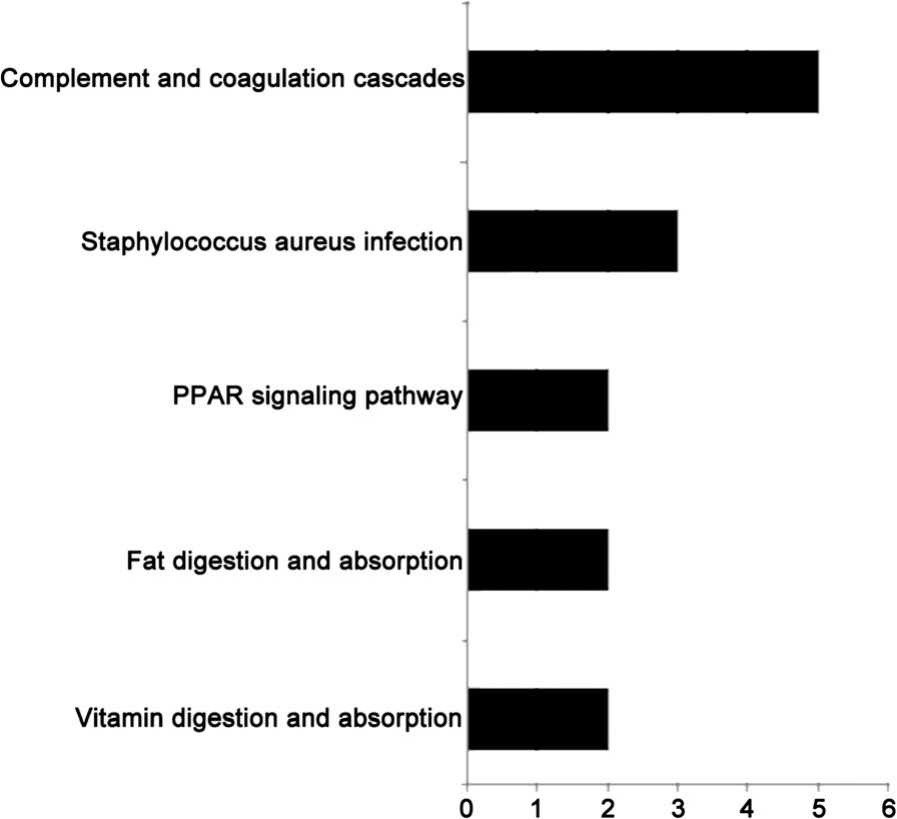
Kegg pathway analysis. The pathways of the differentially expressed proteins indexed by KEGG database (http://www.genome.jp/kegg/)

The KEGG pathway analysis showed that the complement and coagulation cascades were the major pathways in the study. In addition pathways related to *Staphylococcus aureus* infection, peroxisome proliferator-activated receptor (PPAR) signaling, fat digestion and absorption, and vitamin digestion and absorption were also identified (shown in Fig. 2).

**Fig. 3:**
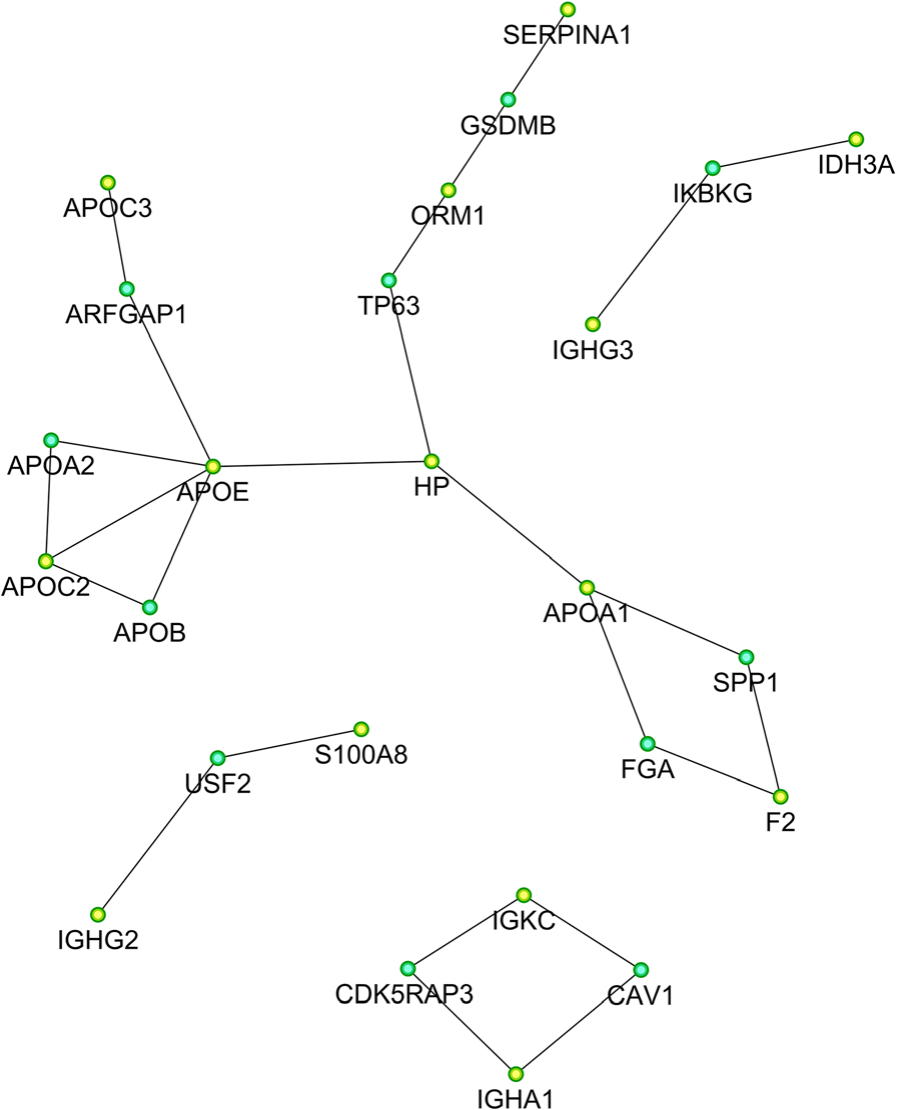
Differentially expressed protein interaction network analysis. Yellow nodes represent target proteins and the green nodes represent the related proteins. SERPINA1, serpin peptidase inhibitor, clade A, member 1; ORM1, orosomucoid 1; HP: haptoglobin; APOA1: apolipoprotein A1; APOB: apolipoprotein B; APOC3: apolipoprotein C3; APOC2: apolipoprotein C2; APOE: apolipoprotein E; GSDMB: gasdermin B; ARFGAP1: ADP-ribosylation factor GTPase-activating protein 1; TP63: tumor protein p63; IGHG3: Ig gamma-3 chain C region; IDH3A: isocitrate dehydrogenase 3 (*NAD+*) alpha; IKBKG: inhibitor of kappa light polypeptide gene; SPP1: secreted phosphoprotein 1; FGA: fibrinogen alpha chain; F2: prothrombin; CAV1: caveolin 1; IGKC: immunoglobulin kappa constant; IGHA1: immunoglobulin heavy constant alpha 1; CDK5RAP3: CDK5 regulatory subunit associated protein 3; USF2: upstream transcription factor 2; IGHG2: immunoglobulin heavy constant gamma 2

To further analyze the 26 differently expressed proteins using STRING, a functional associated network was constructed (shown in Fig. 3). This indicated that APOE was employed in the central role in the LCPD network.

### Western blotting validation

**Fig. 4:**
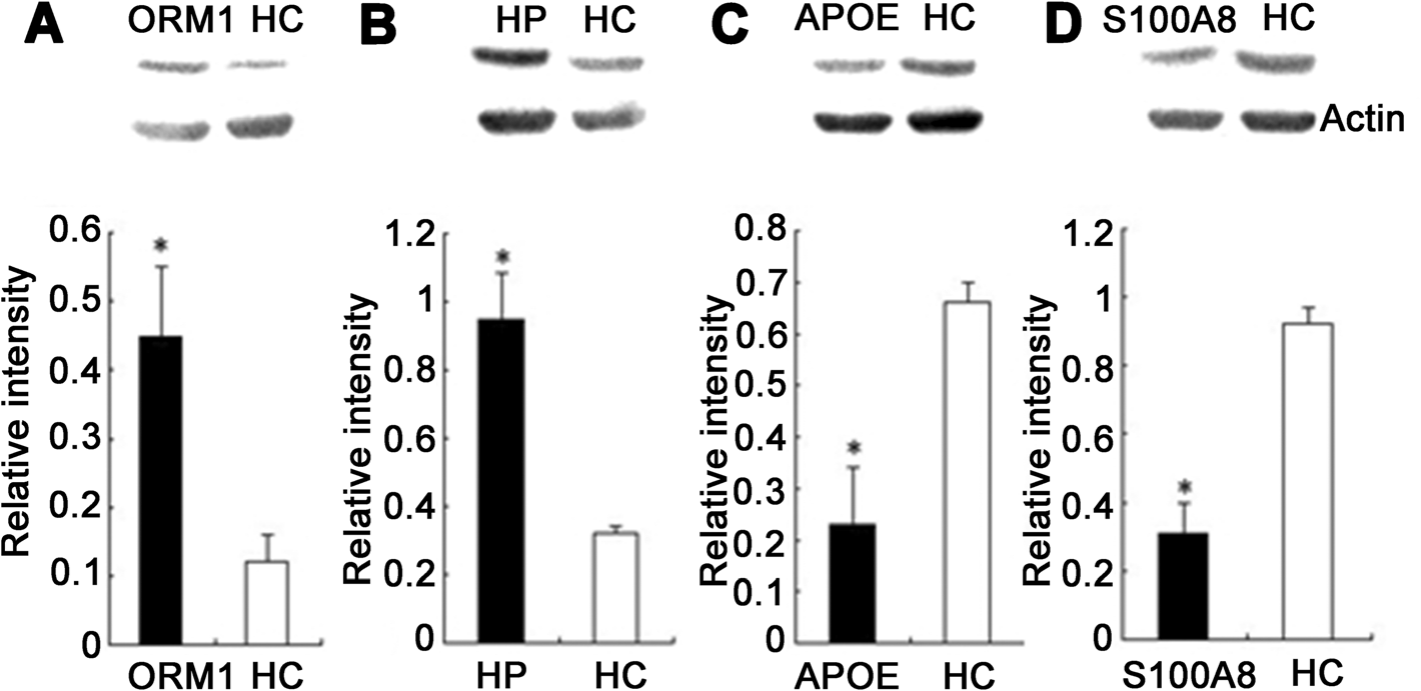
Western blot analysis of four differentially expressed proteins. Relative intensities of the positively identified proteins are shown by the histograms. Patients with Legg-Calve-Pathes disease (*LCPD*) displayed higher levels of **a** orosomucoid 1 *(ORM1*) and **b** haptoglobin (*HP*), and lower levels of **c** apolioprotein E (*APOE*) and **d** S100-A8 than healthy controls (*HC*). The relative density was calculated by dividing the density of matched spot by the density of all the matched spots in the respective gel. *indicates significant difference from the healthy volunteer group, *p* < 0.05

Western blot analysis of four differentially expressed proteins (S100-A8, haptoglobin, alpha-1-acid glycoprotein 1 and apolipoprotein E) was used to verify our mass spectrometry results. They were selected for investigation based on their interesting biological functions and high fold-changes, as well as the availability of commercial antibodies. ORM1 and HP were increased in patients with LCPD, APOE and S100-A8 were decreased in patients with LCPD (shown in Fig. 4). Thus, western blotting was consistent with the results of the mass spectrometry analysis.

## Discussion

In this study, we compared serum proteome expression in LCPD in a Chinese Hans population, and found some prominently differentially expressed proteins in the LCPD patients compared with the healthy controls. The differential serum proteomes provide new-insights into the systemic expression of many reactions that may have occurred in the tissues of patients with LCPD.

Abnormal coagulation has been shown to be closely related with the occurrence of LCPD [4–6]. In our study we found that prothrombin, which is important for blood coagulation, had a different expression level, which is consistent with the previous studies [4–6].

A recent study reported that abnormalities of vascular structure and function occur in children with LCPD [10]. We found nicotinamide adenine dinucleotide (NAD) expression was suppressed in LCPD. Activation of NAD can prevent atherosclerosis and arterial restenosis after vascular injury by suppressing vascular smooth muscle cell proliferation [23].

An immune response is involved in the pathogenesis of many diseases, including femoral head necrosis in adult patients [24]. A previous study showed that genetic variants of interleukin-6 were related with the occurrence of the LCPD [8, 25]. We observed that immune response related proteins were upregulated in LCPD, revealing that an immune response may be involved in this disease. These proteins may also be involved in complement activation.

The LCPD patients showed higher abundance of complement C4-B and complement factor H, which are involved in prohibition of complement activation. Complement activation is supposed to involved in osteoarthritis pathogenesis [26, 27] and adult femoral head necrosis [15]. Therefore, we supposed that cartilage destruction was involved in the pathogenesis of LCPD [28–30]. Very few studies have focused on the pathological role of complement factors in regulating LCPD. Complement factors reportedly control the clearance of necrotic or apoptotic cells in tissue remodeling. The potent biological role of complement factor H in LCPD is unclear. In our study, changes in the abundances of complement factor C presumably indicated disturbances in the homeostasis of inflammatory, necrotic, or apoptotic reactions in LCPD.

S100 proteins are low molecular weight (9 to 14 kDa) intracellular calcium-binding proteins that control key cellular pathways. Chondrocytes and osteoblasts have been shown to express S100-A8 and S100-A9. Both S100-A8 and S100-A9 may contribute to calcification of the cartilage matrix and its replacement with trabecular bone, and redox regulation during bone resorption [31]. They have a possible role in cartilage repair or inflammation-induced degradation [32]. Protein S100-A8, is supposed to be involved in the pathology of osteoarthritis [33, 34], and to have a close relationship with rheumatoid arthritis. Serum levels of S100-A8/S100-A9 were found to correlate better with disease activity and joint destruction in various inflammatory arthritis than classical markers of inflammation [35, 36]. However, both S100-A8 and S100-A9 were decreased in the serum of LCPD patients, showing that LCPD has a different pathogenesis to osteoarthritis.

The functional network of the differently expressed proteins showed that 10 proteins, including serpin peptidase inhibitor, clade A, ORM1, HP, transferrin, APOA1, APOB, APOC3, APOC3, APOE, and APOF, were closely connected by relatively thick lines. Based on the KEGG analysis, these proteins are functionally related to lipid metabolism. Apolipoprotein genes have been repeatedly associated with osteonecrosis [37, 38]. The availability of multiple lines of evidence further strengthens the validity of the relationship between lipid metabolism and LCPD.

Actually, abnormal lipid metabolism has been considered to be an indicator of clinical risk for osteonecrosis and LCPD [39, 40]. It has been shown that lipid metabolism disorders are related to the occurrence of the disease [41]. Furthermore, a recent study reported that leptin played an important role in LCPD pathogenesis [9]. Genetic polymorphisms involved in lipid metabolism were also associated with osteonecrosis of the femoral head in Chinese patients [42]. Taken together, these results suggest that abnormal lipid metabolism may play an important role in the pathology of LCPD.

This study has some limitations. We performed the proteomic analysis on two quite small study populations, although we did verify four differentially expressed genes in a larger population. Further analysis of these results in larger study populations from different ethnic groups would provide more weight to the results. We also did not perform any investigation into the mechanisms suggested by this study. The findings of the present study are the first step in elucidating the proteomic regulation of LCPD. Further Studies will functionally characterize the biological role of the candidate proteins in modulating the development of LCPD. Those details will have to be revealed in later studies.

## Conclusion

Our serum proteomic observations suggest that multiple pathological reactions may occur in the development of LCPD. We have characterized the expression profile and, by extrapolation, defined the functional relationship of a specific set of proteins that were differentially expressed between the LCPD and control groups. The bioinformatics analyses especially highlighted the importance of abnormal lipid metabolism to the occurrence of LCPD.

## Abbreviations

LCPD: Legg-Calve-Perthes Disease
iTRAQ: Isobaric tags for relative and absolute quantification
SDS: Sodium dodecyl sulfate
DTT: Dithiothreitol
pAGC: Predictive automatic gain control
FASP: Filter aided sample preparation
FDR: False discovery rate
GO: Gene ontology
STRING: Search Tool for the Retrieval of Interacting Genes
SERPINA1: Serpin peptidase inhibitor, clade A, member 1
ORM1: Orosomucoid 1
ALB: Albumin
HP: Haptoglobin
TF: Transferrin
APOA1: Apolipoprotein A1
APOB: Apolipoprotein B
APOC3: Apolipoprotein C3
APOC2: Apolipoprotein C2
APOE: Apolipoprotein E
APO: Apolipoprotein F
KEGG: Kyoto encyclopedia of genes and genomes
NAD: Nicotinamide adenine dinucleotide
